# Association Between Helicobacter pylori Infection and Serum Iron Profile

**DOI:** 10.7759/cureus.17925

**Published:** 2021-09-13

**Authors:** Gehna Kishore, Mishal Ejaz, Jatender Kumar, Amar Lal, Hamza Tahir, Zauraiz Anjum, Sidra Naz, Waseem Maher, Sidrah Khan, Amber Rizwan

**Affiliations:** 1 Internal Medicine, Jinnah Sindh Medical University, Karachi, PAK; 2 Internal Medicine, Ziauddin University, Karachi, PAK; 3 Haematology, University of Arizona, Tucson, USA; 4 Internal Medicine, Allama Iqbal Medical College, Lahore, PAK; 5 Internal Medicine, Fatima Jinnah Medical University, Lahore, PAK; 6 Internal Medicine, University of Health Sciences, Lahore, PAK; 7 Internal Medicine, Ghulam Muhammad Mahar Medical College, Karachi, PAK; 8 Internal Medicine, Jinnah Postgraduate Medical Centre, Karachi, PAK; 9 Family Medicine, Jinnah Postgraduate Medical Centre, Karachi, PAK

**Keywords:** h pylori infection, ferritin, iron profile, serum iron level, tibc

## Abstract

Introduction: Helicobacter pylori (H. pylori) infection is reported to be the most frequent cause of morbidity and mortality in cases of upper gastrointestinal (GI) diseases. There is paucity of research between the possible association of H. pylori and iron stores and iron deficiency anemia (IDA). In this study, we will determine if there is an association between serum total iron-binding capacity (TIBC), serum iron and ferritin levels, and H. pylori infection.

Methods: This case-control study was conducted in the gastroenterology ward of a major hospital in Pakistan from December 2020 to April 2021. Three hundred patients diagnosed with H. pylori were enrolled along with 300 participants in the control group. H. pylori was confirmed or excluded with the help of Giemsa stained gastric biopsy specimens. Blood was sent to the laboratory to test for ferritin, serum iron, and TIBC. Each sample was drawn in the morning to avoid any fluctuations.

Results: The mean serum iron level was significantly lower in participants with H. pylori infection compared to those who did not have H. pylori infection (110.72 ± 28.38 ug/dL vs. 162.5 ± 21.18 ug/dL; p-value: <0.0001). Serum ferritin level was significantly higher in participants with H. pylori infection (536.82 ± 117.0 ng/dL vs. 391.31 ± 101.54 ng/dL; p-value: <0.0001).

Conclusion: In comparison with the control group, TIBC and serum iron levels were found to be lower in the case group.

## Introduction

Helicobacter pylori (H. pylori), a spiral-shaped gram-negative bacterium, is known to infect the stomach. The infection is prevalent in approximately 80% of the world’s population, which is why it is the most common infection in humans. The prevalence rate is particularly higher in developing countries with a rough idea of it being 80% [1]. This pathogen is positively correlated with a number of gastrointestinal (GI) diseases like type B antral gastritis, peptic ulcer, gastric mucosa-associated lymphoid tissue lymphoma, and gastric adenocarcinoma [2]. Potential risk factors include unsatisfactory hygiene, dense population, intake of undercooked food from streets, and impure water availability [3]. Another risk factor that increases the chance of getting H. pylori infection is smoking and smokeless tobacco. This is highly prevalent in South Asian countries, particularly Pakistan [4,5].

In the case of upper GI diseases, H. pylori infection is reported to be the most frequent cause of morbidity and mortality. Recent studies have demonstrated the effects of H. pylori in causing extra-GI diseases, namely coronary disease, myocardial infarction, Raynaud’s syndrome, migraine, dermatological disorders, iron deficiency anemia (IDA), and some autoimmune diseases [6]. The effects of H. pylori infection on iron reserves (ferritin) and iron deficiency were linked in a 2013 research of metropolitan Alaska native adults. According to the findings, eradication therapy for H. pylori can simultaneously raise blood ferritin levels, thereby resolving iron deficiency because there will be greater iron stores. Another study published in 2014 showed the need for a healthy GI tract (GIT) for iron absorption. As a result, the study indicated that people with normal GIT mucosa and H. pylori infection did not have iron deficiency, whereas people with abnormal GIT mucosa and H. pylori infection did [7].

There is limited regional data available to study the correlation between H. pylori infection and serum iron levels. In this study, we will determine if there is an association between serum total iron-binding capacity (TIBC), serum iron and ferritin levels, and H. pylori infection.

## Materials and methods

This case-control study was conducted in the gastroenterology ward of a major hospital in Pakistan from December 2020 to April 2021. We enrolled 300 patients diagnosed with H. pylori, between the ages of 18 and 50 years, and of either gender. Participants were explained the entire protocol and their consent was taken. They were enrolled via consecutive convenient non-probability sampling. Institute approval was taken before the start of the study (JSMU/IRB/2020/31). Another 300 participants, adjusted for age and gender, without a diagnosis of H. pylori were included in the study as a case group. Women menstruating, participants on iron supplements, or suffering from chronic diseases like kidney disease or malignancy were excluded from the study.

H. pylori was confirmed or excluded with the help of Giemsa stained gastric biopsy specimens. A blood sample of 5 ml was drawn from the cubital vein from both the case and control groups and sent to the laboratory to test for ferritin, serum iron, and TIBC. Each sample was drawn in the morning to avoid any fluctuations.

The Statistical Packages for Social Sciences (SPSS) v. 21.0 was used for statistical analysis (IBM Corp., Armonk, NY). The mean and standard deviation of continuous variables were calculated using descriptive statistics. Percentages and frequencies were used to present categorical variables. An independent t-test was used to compare serum ferritin and iron levels, and TIBC between the two groups. A p-value of less than 0.05 indicated that there was a significant difference between the case and control groups, indicating that the null hypothesis was incorrect.

## Results

There were more male participants in our study, however, the ratio was comparable between both groups. The most common age group was between 41-50 years (Table 1).

**Table 1:** Demographics of the participants NS: nonsignificant

Demographics	Case group (n=300)	Control group (n=300)	p-value
Gender			
Male	161 (53.6%)	159 (53.0%)	NS
Female	139 (46.4%)	141 (47.0%)	
Age Group (in years)			
18-30	51 (17.0%)	57 (19.0%)	NS
31-40	62 (20.6%)	59 (19.6%)	
41-50	109 (36.3%)	111 (37.0%)	
51-60	78 (26.0%)	73 (24.3%)	

The mean serum iron level was significantly lower in participants with H. pylori infection compared to those who did not have H. pylori infection (110.72 ± 28.38 μg/dL vs. 162.5 ± 21.18 μg/dL; p-value: <0.0001). Serum ferritin level was significantly higher in participants with H. pylori infection (536.82 ± 117.0 ng/dL vs. 391.31 ± 101.54 ng/dL; p-value: <0.0001) (Table 2).

**Table 2:** Comparison of the iron profile of case and control groups μg/dL: micrograms per decilitre, ng/dL: nanograms per decilitre, TIBC: total iron-binding capacity

Iron profile	Case group	Control group	p-value
Serum iron (μg/dL)	110.72 ± 28.38	162.5 ± 21.18	<0.0001
Serum ferritin (ng/dL)	536.82 ± 117.0	391.31 ± 101.54	<0.0001
Serum TIBC (μg/dL)	125.81 ± 23.41	141.12 ± 20.99	<0.0001

## Discussion

The current case-control study concluded an inversely proportional relationship between serum iron levels and H. pylori infection. People infected with H. pylori infection have very low serum iron levels. The serum ferritin levels were found to be high in H. pylori-infected individuals, owing to the inflammatory conditions after H. pylori infection, which may lead to the increased levels. However, this is not consistent with a previous study where eradication of H. pylori infection was associated with an increase in serum ferritin levels. Low serum iron levels are highly associated with IDA and effective management of H. pylori infection can overcome anemia and increase iron stores for a better quality of life [8].

Various studies have highlighted the relationship between H. pylori infection and IDA, the most common cause of low serum iron levels, was assessed [9,10]. A study conducted in the United States highlighted that H. pylori infection among individuals was more likely associated with IDA and less likely with other types of anemia. Anemia due to H. pylori might be due to an alteration in iron metabolism [9]. Iron deficiency and IDA occur when there are depleted stores of iron in the body. This could be due to many possible factors; a significant one being peptic ulcers. The study correlated that individuals who were prone to have a history of peptic ulcers were at a greater risk of H. pylori infection and iron deficiency and IDA [9].

Another study conducted by Miernyk et al. also mentioned that 121 out of 241 patients were H. pylori-positive and the majority of infected individuals had IDA compared to the uninfected individuals [8]. This study also correlated the effect of H. pylori eradication treatment to increased serum ferritin levels. It was concluded that H. pylori eradication improved serum ferritin levels [8]. This conclusion differs from our study as serum ferritin levels are increased with ongoing H. pylori infection. Serum ferritin, a significant marker for iron status, may be increased due to inflammatory changes from the H. pylori infection itself [11] and unresponsiveness to iron therapy [12]. The eradication therapy for H. pylori is not only effective for serum ferritin levels [7] but also has a positive effect on other blood parameters, including complete blood count. Hemoglobin (Hb), serum transferrin, and iron levels were increased at 20-24 weeks of follow-up after eradication therapy [13]. IDA is one of the leading causes of death worldwide and about 2 billion people lost their lives worldwide [14]. It occurs when physiological requirements cannot be met by dietary intake of iron alone due to multiple factors [14]. Iron deficiency can stem from a multitude of reasons, such as chronic blood loss, poor intake, chronic diseases, and GI infections, e.g. H. pylori. There is a significant gap in the literature regarding knowledge of pathogenesis for IDA secondary to H. pylori infection [15]. Few studies conducted concluded that individuals with IDA due to H. pylori do not complain of GI symptoms, gastroduodenal hemorrhagic lesions, malnutrition, or poor intake but the only known evidence for pathogenesis was H. pylori-related gastritis. These features may be used as a template for further controlled studies to identify the exact mechanisms of this atypical, medically significant, putative consequence of H. pylori infection [12]. A study conducted by Marshall et al. demonstrated curved bacilli in biopsy of patients with active chronic gastritis, duodenal and gastric ulcers, thus, it may be an important consequence of H. pylori infection [16]. Ulcers usually tend to bleed leading to iron depletion, but, in a study, six patients with IDA did not have hemorrhagic symptoms [16]. Ghosh S in his study linked refractory IDA with H. pylori and autoimmune gastritis [17] Based on our study, patients diagnosed with H. pylori should have their iron profile done to screen for underlying cause for abnormal iron profile. 

To the best of our knowledge, this is the first study in the region that studies the association between H. pylori infection and serum iron profile. However, since all participants were from the same institute, their socio-demographic profiles were similar and the sample size was less diverse. Also, since it was a case-control study, the definite associations between the low iron profile and H. pylori could not be established. Furthermore, the reason for low serum iron was not assessed.

## Conclusions

In comparison with the control group, TIBC and serum iron levels were found to be lower in the case group. Owing to the results of our study, we conclude a positive association between H. pylori and low serum iron. Therefore, in patients with H. pylori, prompt work-up for iron profile should be done. Timely and appropriate eradication treatment along with iron supplementation may lead to the quicker resolution of H. pylori; however, this needs to be explored in further large-scale trials.
